# Ten tips in lupus nephritis management

**DOI:** 10.1093/ckj/sfae376

**Published:** 2024-11-22

**Authors:** Selene T Y Teoh, Desmond Y H Yap, Tak Mao Chan

**Affiliations:** Division of Nephrology, School of Clinical Medicine, Queen Mary Hospital, University of Hong Kong, Hong Kong SAR; Division of Nephrology, Department of General Medicine, Khoo Teck Puat Hospital, Singapore; Division of Nephrology, School of Clinical Medicine, Queen Mary Hospital, University of Hong Kong, Hong Kong SAR; Division of Nephrology, School of Clinical Medicine, Queen Mary Hospital, University of Hong Kong, Hong Kong SAR

**Keywords:** acute kidney injury, adherence, chronic kidney disease, lupus nephritis, systemic lupus erythematosus

## Abstract

Lupus nephritis is an important cause of severe glomerulonephritis, and a leading cause of kidney failure in young adults. While the disease can lead to rapid destruction of nephrons if untreated, there are effective therapies to reverse the severe acute kidney injury and prevent the lifetime risk of kidney failure. Early diagnosis and timely intervention are therefore of critical importance. Clinical management of lupus nephritis has improved considerably over the past two decades. The advent of mycophenolate as standard immunosuppressive therapy was a major paradigm shift that improved the safety and convenience of treatment and also patients' quality of life. Effective therapeutic options continue to increase, such as belimumab (a monoclonal antibody that inhibits B-cell activating factor, BAFF) and voclosporin (a calcineurin inhibitor) which have obtained regulatory approval in U.S.A. and Europe. There is also accumulating experience on tacrolimus, which has regulatory approval for lupus nephritis treatment in Japan and commonly used off-label in many countries. Ironically, the increasing therapeutic options have resulted in uncertainties in deciding which medication, and which treatment regimen, is best for a patient. In this context, one needs to take into consideration the distinct characteristics and the risk profile of each patient, and adopt a holistic and long-term perspective, so that treatment can be personalized to achieve favourable clinical outcomes.

## INTRODUCTION

The management of lupus nephritis (LN), a severe organ manifestation affecting 30–60% of patients with systemic lupus erythematosus (SLE), can be challenging [1]. Over the last few decades, advances in the knowledge on pathogenic mechanisms have brought novel therapies for LN. However, LN remains an important cause of morbidity and mortality [2, 3]. Although patient and kidney survival rates have improved over the years, progressive chronic kidney disease (CKD) is still prevalent, with up to 20% of patients eventually developing end-stage kidney disease (ESKD) [4–6]. Current standard-of-care treatment for active LN, comprising glucocorticoid in combination with mycophenolic acid or reduced-dose cyclophosphamide, with or without belimumab or calcineurin inhibitors (CNIs), achieves combined complete or partial remission rates of ≥70% [7]. However, challenges remain in deciding which drugs to use and what is the best treatment regimen for each patient. The answers to these questions can also change over time in the same patient. Variations in disease severity, timing of presentation and genetic background may affect treatment response, and relapses are common while the risk varies between patients. Rapid abatement of inflammatory kidney injury, consolidating and maintaining a durable remission and preventing flares are vital in preserving nephron mass, preventing CKD progression, complications and death due to infections, cardiovascular disease and malignancies. Achieving treatment targets needs to be delicately balanced against the risks of drug toxicity and overimmunosuppression. It is also important to consider the burden of disease and treatment and their impact on fertility, pregnancy, psychosocial and economic aspects and quality of life.

## TIP 1: SEVERITY OF ACTIVE AND CHRONIC KIDNEY HISTOPATHOLOGICAL LESIONS INFORMS CHOICE OF MEDICATIONS AND DOSAGE, WHILE EXPEDITIOUS TREATMENT IS ESSENTIAL TO STOP ONGOING NEPHRON DAMAGE

LN typically presents with haematuria, increasing proteinuria or increasing serum creatinine, alone or in combination. The kidney biopsy, in addition to confirming the diagnosis of LN, provides valuable information on activity, chronicity and severity. Classes III and IV of the International Society of Nephrology/Renal Pathology Society (ISN/RPS) classification are the most severe types of active LN, with immune-mediated acute injury in the mesangium and glomerular capillaries, often associated with tubulointerstitial inflammation, which can rapidly cause irreversible nephron damage [8]. Early diagnosis and expeditious treatment are essential to ensure optimal outcome [9, 10]. Severe active LN should be managed akin to a medical emergency to prevent the accumulation of irreversible damage to nephrons. Delayed diagnosis has been shown to be associated with poor kidney outcomes [11, 12]. In one study, delayed diagnostic kidney biopsy for >6 months after onset of nephritis symptoms was associated with 9-fold higher risk of ESKD [12].

The relationship between histological LN class alone and long-term kidney survival is inconsistent [11, 13–15], due to confounding factors such as response to treatment and the extent of irreversible damage. Active and chronic histopathological lesions provide important information on severity, reversibility and prognosis. Semiquantitative activity or chronicity indices are useful, but the scoring requires experience and is fraught with intra- and interobserver variation [16–18]. Endocapillary hypercellularity can be reversible with therapy, while extensive crescents warrant more aggressive therapy. Extensive chronic lesions such as interstitial fibrosis and tubular atrophy portend unfavourable renal prognosis. Additional lesions not included in the current ISN/RPS classification should be noted, as these could indicate specific pathogenic autoantibodies or processes that require special management. Different types of vascular lesions may occur in LN and their presence has prognostic and treatment implications [19, 20]. For example, thrombotic microangiopathy (TMA) may occur in isolation or with glomerular proliferative lesions and is associated with more severe clinicopathological features and worse renal outcomes compared with patients with proliferative lesions only [21, 22]. TMA may be associated with anti-phospholipid antibodies or ADAMTS-13 autoantibodies and treatment considerations include anticoagulation and plasma exchange [21, 23]. Nephrotic syndrome in association with extensive podocyte foot process effacement, characteristic of lupus podocytopathy, can be associated with class I/II LN [24, 25]. While it is often steroid-responsive, there is a significant risk of disease relapse [7]. Apart from active nephritis, proteinuria and increased serum creatinine can be due to other factors, including CKD, hypertension, dietary protein intake, fluid status and other causes of acute kidney injury [26]. When in doubt whether a patient has unresolved or resurgent active nephritis, a repeat kidney biopsy is useful.

## TIP 2: NOTE PRIOR HISTORY OF DISEASE FLARES AND TREATMENT RESPONSE OR TOLERANCE WHEN DECIDING ON THE OPTIMAL TREATMENT REGIMEN

The disease course and response to treatment can vary considerably between patients. Patient characteristics such as age, genetic background, clinical phenotype at disease onset, extrarenal manifestations, duration and severity of disease all impact risk profiling and drug choice. The number and severity of previous flares also indicate previous drug exposure, subsequent risk of toxicities and damage accrual. It is important to note the prior response and tolerance to various immunosuppressive drugs and regimens. Patients who have received B cell–depleting agents need to be monitored for late-onset neutropenia and hypogammaglobulinaemia [27]. SLE is a relapsing disease, but the risk of developing flares varies considerably between patients. Maintenance immunosuppression prevents flares, with mycophenolate acid (MPA) being superior to azathioprine and is recommended in current guidelines in combination with an antimalarial [7, 28]. Current guidelines recommend at least 3 years of maintenance immunosuppressive treatment based on available data, but in practice many patients continue with low-dose immunosuppressive medication(s) for many more years. Uncertainty remains with regard to the optimal duration of maintenance immunosuppressive therapy, which is likely to vary between patients in view of differences in genetic background and flare risk. In a French multicentre trial (Weaning of Maintenance Immunosuppressive Therapy in Lupus Nephritis), LN patients who discontinued immunosuppression after 2–3 years of maintenance developed significantly more renal flares (27.3% versus 12.5%) and more severe flares than those who continued on treatment [29]. Another study in SLE patients (38% with LN) with clinically inactive disease for at least 1 year showed that discontinuation of glucocorticoid was associated with increased risk of flare [30]. Overall, a gradual tapering schedule and a risk–benefit approach should guide decisions on weaning immunosuppression. The dose of immunosuppressive medications at which flares occur in a patient informs decisions on tapering and discontinuation of drugs in subsequent management. An individualized approach is required and repeat kidney biopsy to identify subclinical histological activity may be useful in selected patients [31].

## TIP 3: AVOID EXCESSIVE EXPOSURE TO GLUCOCORTICOID AND OTHER IMMUNOSUPPRESSIVE DRUGS TO PREVENT SHORT- AND LONG-TERM TOXICITIES

High-dose glucocorticoid has been used as treatment for severe SLE and LN since the 1960s. There is increasing evidence that with the current combined immunosuppressive treatment regimens, it is feasible to reduce glucocorticoid exposure without sacrificing treatment efficacy. Presently, initial treatment for severe LN typically starts with daily intravenous methylprednisolone pulses of ≈500 mg for 2–3 days, followed by oral prednisolone with progressive tapering. The glucocorticoid dose and tapering regimen vary between clinicians. Starting at a daily prednisone dose of ≤0.8 mg/kg is generally accepted, and many clinicians start at a lower dose of 0.5–0.6 mg/kg/day following pulse methylprednisolone [32–34]. The recent European League Against Rheumatism (EULAR) guidelines recommend prednisone at 0.3–0.5 mg/kg/day following pulse intravenous methylprednisolone, although this recommendation is largely opinion based [28]. A recent meta-analysis of 50 protocolized glucocorticoid arms from randomized controlled trials involving >3200 patients showed a dose–response relationship between initial glucocorticoid dose and rates of complete remission, serious infections and death at 6 months, and a disproportionate increase in the risk of serious infections and death was noted in patients taking >40 mg/day (>0.6–0.7 mg/kg/day) of prednisone, especially when pulse steroid was also given [35]. While higher steroid doses are more effective in treating active nephritis, it should be tailored to disease severity and excessive glucocorticoid exposure should be avoided to prevent infections and other steroid-related adverse effects. Rapid tapering of the oral prednisone dose to 2.5 mg/day at week 16 was adopted in the voclosporin LN trials, but the long-term results with this regimen remain to be established [36, 37]. A daily prednisone dose of ≤5 mg is recommended for stable patients in the maintenance phase [7, 38, 39]. Recent data have shown that belimumab added to standard therapy improved treatment responses and reduced flare rates [40–42] and allowed for glucocorticoid dose reduction in a few real-world cohorts [43–45]. Persistent proteinuria, more common in patients with membranous changes, could benefit from addition of a CNI, given its stabilizing effect on podocyte cytoskeleton [46]. CNIs have demonstrated efficacy and are increasingly used in the treatment of LN. Adding voclosporin to glucocorticoid and mycophenolate resulted in more effective reduction of proteinuria [36], while tacrolimus was shown to be effective in reducing persistent proteinuria in patients with membranous LN [47, 48]. In addition to other side effects such as hypertension, dyslipidaemia and diabetogenesis, it is important to avoid excessive CNI exposure in order to prevent CNI-induced nephrotoxicity, especially in patients with CKD.

## TIP 4: TREAT RISK FACTORS TO PREVENT ADVERSE CARDIOVASCULAR AND METABOLIC OUTCOMES

Cardiovascular risk factors, such as hypertension, hyperlipidaemia and diabetes mellitus, are common in patients with LN and are important contributors to cardiovascular morbidity and mortality [49]. The burden of these risk factors increases over time, due to aging, progressive CKD and treatment-related adverse effects. The EULAR recommendations for management of SLE acknowledge that generic cardiovascular risk assessment scores, such as the Framingham Risk Score, underestimate cardiovascular risk in SLE patients [50, 51]. Newer cardiovascular risk prediction tools have been reported, but require further validation [52]. The mechanisms of accelerated atherogenesis in SLE are complex, and disease-specific factors such as a pro-inflammatory milieu and endothelial dysfunction may contribute to excess cardiovascular risk [53]. Traditional cardiovascular risk factors and metabolic side effects of drugs such as glucocorticoid and CNI should be monitored on a regular basis. Hypertension should be treated with renin–angiotensin system (RAS) blockers as the first choice for antihypertensive therapy [50]. Treatment for hyperlipidaemia should include dietary control and statin therapy [54]. Hydroxychloroquine use was associated with reduced organ damage accrual and cardiovascular events and is therefore recommended for all patients, even when the patient is treated with other immunosuppressive medications, with regular surveillance for retinal toxicity as recommended in current guidelines [7, 50]. The timing of cardiovascular investigations for early identification of coronary artery disease remains uncertain, but should be considered in high-risk patients such as those with persistent proteinuria, uncontrolled hyperlipidaemia or a family history of coronary artery disease of early onset. Smoking is prohibited, as it is detrimental to vascular and kidney health.

## TIP 5: PREVENT INFECTIVE COMPLICATIONS DUE TO IMMUNOSUPPRESSION

Infection is a leading cause of death in SLE patients and is an important cause of mortality and morbidity in patients with LN [55, 56]. The risk for infection is highest after exposure to intensive immunosuppression for disease flares (the ‘induction’ phase), although severe infections can still occur in patients with quiescent disease on maintenance immunosuppression [57]. While immunosuppression needs to be given at an adequate dose for rapid control of disease, it is important to avoid overimmunosuppression, as risks of opportunistic infections increase with increasing immunosuppressive burden. *Pneumocystis jirovecii* pneumonia (PJP) is a well-recognized opportunistic infection and is associated with unfavourable outcomes [58–60]. PJP prophylaxis must be included when prednisone exposure is >20 mg/day for >4 weeks or when glucocorticoid is used in combination with other immunosuppressive drugs [7, 61, 62]. The infective microorganisms and preventive strategies also take into consideration the epidemiology of pathogens. In areas where hepatitis B virus (HBV) is prevalent, such as the Asia–Pacific regions, it is mandatory to screen patients for viral markers to assess the need for preventive antiviral therapies [61]. In this context, antiviral prophylaxis should be given to patients with occult hepatitis B infection (i.e. hepatitis B surface antigen negative but anti-hepatitis B core antigen positive), especially those receiving anti-CD20 treatment [63]. The risk of tuberculosis needs to be considered. Relevant screening tests (e.g. chest X-ray or interferon gamma release assays) should be performed and prophylaxis administered according to local guidelines. The optimal duration of anti-infective prophylaxis remains undefined and should take into consideration the type, dose and duration of immunosuppressive therapy in combination with host risk factors, such as age and comorbidities. Vaccinations such as those for influenza and pneumococcus are recommended, but live vaccines must be avoided (Table 1). Patients with SLE and LN are at increased risk of developing herpes zoster, especially following exposure to high-dose glucocorticoid with MPA or CNI [64–66]. There is preliminary data showing that the recombinant Shingrix vaccine is safe for the prevention of zoster reactivation in SLE patients [67].

**Table 1:** Mitigating the risk of infections when managing patients with LN.

	Screening	Prophylaxis	Vaccination
Standard of care applicable to all patients	Hepatitis B virus Hepatitis C virus Human immunodeficiency virus	Opportunistic infections, e.g. *Pneumocystis jirovecii* Reactivation of latent infections: hepatitis B virus, tuberculosis	Hepatitis B Influenza *Streptococcus pneumoniae* COVID-19
In specific cases or with relevant epidemiologic exposure	Tuberculosis Syphilis and other STIs	Encapsulated organisms (in patients receiving complement inhibitors)	Shingrix^®^ in patients at risk of zoster reactivation Encapsulated organisms (in patients receiving complement inhibitors)

COVID-19: coronavirus disease 2019; STIs: sexually transmitted infections.

## TIP 6: NOTE THE SEVERITY OF UNDERLYING CHRONIC KIDNEY DISEASE AND RENAL RESERVE SINCE THEY IMPACT A PATIENT'S RISK PROFILE AND INFORM THE CHOICE OF THERAPY

CKD of variable severity is common in patients with LN, as inflammation in active nephritis destroys nephrons and produces scars in the kidney [68]. CKD is easily underrecognized in patients with LN, as the serum creatinine level may remain within the ‘normal’ range even when there is significantly impaired kidney function, especially in Asian female patients with small body size and muscle mass [69]. Reported CKD rates of 10–50% might have underestimated the true prevalence since many reports define CKD as an estimated glomerular filtration rate <60 ml/min/1.73 m^2^ [11, 70–80]. Nephritis flares, treatment non-adherence, hypertension and proteinuria contribute to progressive CKD. The presence and severity of CKD have major implications on the choice of therapy, optimal drug dosage and susceptibility to treatment toxicities. For example, CNI should be avoided in patients with severe kidney impairment unless absolutely necessary, and a lower CNI exposure with regular therapeutic drug-level monitoring is necessary in patients with CKD to minimize the risk of CNI nephrotoxicity. Patients with CKD are more susceptible to the marrow suppressive effect of MPA, and therapeutic drug monitoring has a role in dosage optimization in patients with CKD who are treated with mycophenolate [7]. A patient's renal reserve also has an important bearing on decisions regarding the rate of tapering or discontinuation of maintenance immunosuppression, as another nephritis flare would markedly accelerate progressive CKD. Also, renoprotective medication(s) should be included in the treatment of patients with CKD, including RAS blockers [68] and possibly sodium–glucose co-transporter-2 inhibitors [81, 82], non-steroidal mineralocorticoid receptor antagonists [83, 84] and glucagon-like peptide-1 agonists [85, 86].

## TIP 7: A LONG-TERM AND HOLISTIC PERSPECTIVE IN PATIENT MANAGEMENT IS NECESSARY TO MINIMIZE THE LIFETIME RISK OF KIDNEY FAILURE, PREMATURE DEATH AND OTHER ADVERSE OUTCOMES

SLE is a disease that affects predominantly young individuals, with 10–20% presenting during childhood [87]. Prevention of the lifetime risk of kidney failure and premature death and maintaining a high quality of life with minimum morbidities are the ultimate goals. Clinical management decisions should therefore incorporate these long-term perspectives to prevent progressive CKD, maintain cardiovascular health, preserve fertility, maintain bone health and minimize the risk of malignancy.

As highlighted in Tip 6, nephritis flares result in progressive reduction of nephron mass and increase the lifetime risk of kidney failure. Prevention of flare is therefore critically important in patients with CKD and a history of flares. Treatments with proven efficacy in this regard, such as mycophenolate and belimumab, would be pertinent. Cyclophosphamide treatment is associated with permanent azoospermia and primary ovarian failure in men and women, respectively [88]. Attention to lifetime cumulative cyclophosphamide dose is important to reduce the risk of gonadal toxicity and malignancies, the former especially in older women who have lower oocyte reserve [89]. In this regard, the reduced-dose Euro-Lupus cyclophosphamide regimen results in lower drug exposure [90]. Fertility preservation methods, such as gonadotropin releasing hormone analogues in menstruating women or cryopreservation of oocytes or sperm should be considered [91]. Women of child-bearing age should receive appropriate counselling for contraception when given teratogenic medications such as cyclophosphamide and mycophenolate [91]. Men receiving cytotoxic therapy should avoid conception for at least 3 months after dosing. Pregnancy in women with SLE, particularly those with LN, is associated with increased risks of pre-eclampsia, foetal growth restriction, foetal loss and preterm delivery [92, 93]. These risks are present even during quiescent disease and are higher in patients with hypertension or proteinuria [94, 95]. Good preconception counselling and planning increase the probability of successful pregnancies and reduce risk of pregnancy complications [88]. As lupus activity is a key predictor of adverse pregnancy outcome [93, 96], pregnancy should be avoided until at least 6 months after disease quiescence is achieved. Patients receiving mycophenolate maintenance may switch to azathioprine or CNI and should be observed to ensure stable disease after switching before conceiving [97, 98]. Neoplasm is an important cause of death in LN patients [3], with examples such as non-Hodgkin's lymphoma, lung and breast cancers [99, 100]. Exposure to immunosuppression, especially cytotoxic medications such as cyclophosphamide, are documented risk factors.

Long-term bone health is a common concern in LN patients, as they receive high-dose corticosteroids during induction and a prolonged course of corticosteroids during maintenance [101]. Osteoporosis and osteopenia are common, increasing the risks of peripheral and vertebral fractures, which negatively impact quality of life and increase morbidity and mortality [102]. Adequate calcium and vitamin D supplementation and minimizing the dose and duration of steroid therapy are important preventive measures. There is little consensus on the use of anti-resorptive therapy in LN patients and decisions should be individualized according to the bone densitometry trend, cumulative glucocorticoid dose, presence of established osteopenia or osteoporosis, history of fragility fractures and level of renal function. Of note, children and adolescents with SLE are at higher risk of developing osteoporosis, as they tend to have a high cumulative glucocorticoid exposure before peak bone mass is achieved [103]. This subset of patients with early-onset SLE typically has a more aggressive clinical phenotype and disease course, with a greater burden of chronic damage in the kidneys and musculoskeletal and neurological systems [104]. Both disease and treatment-related complications, including effects on growth, pubertal development and fertility, must be addressed early and not overlooked in their transition to adult nephrology care [105].

## TIP 8: JOINT CARE WITH RHEUMATOLOGISTS AND OTHER HEALTHCARE PROFESSIONALS TO ADDRESS NON-RENAL AND PSYCHOSOCIAL ISSUES AND QUALITY OF LIFE

LN patients frequently experience various extrarenal manifestations. Patients with substantial symptom burdens or severe extrarenal disease should be managed jointly with a rheumatologist and other relevant specialties, due to the complexities in treatments [28]. On the other hand, LN patients under the care of rheumatologists can benefit from joint care with nephrologists, especially those with significant CKD and/or suspected nephritis flare [106]. The overall quality of care may also be improved by involving other allied healthcare professionals such as specialty nurses, clinical psychologists, social workers or pharmacists who can help enhance patient education and address any psychosocial barriers to treatment [107].

## TIP 9: ENSURE TREATMENT ADHERENCE BY BUILDING A STRONG RAPPORT

Non-adherence to treatment is prevalent in SLE [108] and contributes to disease relapse, higher acute care utilisation, hospitalisations and adverse patient outcomes [109, 110]. Factors pertinent to poor treatment adherence include low socio-economic status or health literacy, patient misconceptions and significant treatment-related side effects [111, 112]. Cosmetic and other side effects from medications are common concerns. Building rapport and ensuring patient adherence to treatment is a continuous effort in the course of managing patients with LN, which spans many decades. Compliance should be assessed during follow-up and reasons for non-adherence should be addressed through open and non-judgemental discussions [113]. Measurement of medication blood levels such as MPA and CNI can be helpful to ensure adherence to treatment. Because of its long terminal elimination half-life (7–40 days), unscheduled measurement of whole-blood hydroxychloroquine levels can be used to identify patients who take the medicine only shortly before a follow-up appointment [111]. Frank discussions of treatment priorities with empathy and attention to patient preferences and concerns are essential.

## TIP 10: INDIVIDUALIZE CLINICAL MANAGEMENT TO SUIT A PATIENT'S DISTINCT CHARACTERISTICS AND RISK PROFILE

Management of LN is complex because each patient is different, with a different genetic background, risk profile, drug pharmacogenomics and pharmacokinetics and psychosocial perspective. Individualization of LN management takes into consideration each patient's unique characteristics to decide on treatments that can improve short- and long-term clinical outcomes. While the choice of therapy for active LN is informed by evidence from clinical trials, one must be mindful of the inclusion and exclusion criteria and patient demographics in those trials and consider the distinct characteristics and unique risk profile of each patient in the real-world setting. Increased therapeutic options with proven efficacy such as belimumab and CNI and potential new treatments such as Obinutuzumab, which targets B lymphocytes, and anifrolumab, which targets type I interferon, would facilitate personalized clinical management.

## CONCLUDING REMARKS

While LN remains a complex disease, recent advances in clinical therapeutics have provided increasing options and approaches in clinical management. Novel therapies are changing the treatment landscape in LN, with positive results from some and various agents targeting different immune pathways under study. Consideration of a patient's risk profile and assessment of unique clinical and renal histopathological features can guide individualized management and personalized care, with the objective of achieving optimal long-term kidney and patient survival and quality of life with minimal morbidities and complications (Table 2).

**Table 2:** A summary of practical tips in LN management.

**Tip 1. Severity of active and chronic kidney histopathological lesions informs choice of medications and dosage, while expeditious treatment is essential to stop ongoing nephron damage** Kidney biopsy provides information on activity, chronicity, severity, and specific histopathologic lesions, that guide treatment decisions. Expedite investigations and early treatment to stop ongoing immune-mediated nephron loss.
**Tip 2. Note prior history of disease flares and treatment response or tolerance when deciding on optimal treatment regimen** Natural history and risk of flares can vary considerably between patients. Pay attention to previous disease course and complications, including drug intolerance and risks of cumulative toxicity from previous treatment.
**Tip 3. Avoid excessive exposure to glucocorticoid and other immunosuppressive drugs to prevent short- and long-term toxicities** Combined immunosuppressive regimens improve treatment efficacy and facilitate a reduction of glucocorticoid exposure. Effective prevention of disease flares reduces lifetime exposure to glucocorticoid.
**Tip 4. Treat risk factors to prevent adverse cardiovascular and metabolic outcomes** Be cognizant of cardiovascular and metabolic risks associated with disease and treatment. Investigate for pre-symptomatic cardiovascular disease in high-risk patients.
**Tip 5. Prevent infective complications due to immunosuppression** Screen for chronic or latent infections prior to commencing immunosuppression. Prophylaxis against common opportunistic infections, specific infections associated with treatments and reactivation of latent infections. Vaccination before immunosuppression.
**Tip 6. Note the severity of underlying chronic kidney disease and renal reserve since they impact a patient's risk profile and inform choice of therapy** Presence of CKD impacts drug choice, optimal dosage, and susceptibility to toxicities. Severity of CKD is related to risk of kidney failure and impacts decisions on the tempo of weaning immunosuppression. Incorporate renoprotective strategies and treatments to protect renal reserve.
**Tip 7. A long-term and holistic perspective in patient management is necessary to minimize the lifetime risk of kidney failure, premature death and other adverse outcomes** Consider the impact on disease course and long-term health issues (e.g. fertility and reproduction, bone health, malignancy and cardiovascular risks) when selecting immunosuppressive drugs. Recognize and address the higher burden of physical and psychosocial complications in patients with childhood-onset SLE.
**Tip 8. Joint care with rheumatologists and other healthcare professionals to address non-renal and psychosocial issues and quality of life** Multidisciplinary care to address extrarenal and other health or psychosocial issues.
**Tip 9. Ensure treatment adherence by building a strong rapport** Treatment non-adherence is a well-established cause for unsatisfactory response and patient outcomes.
**Tip 10. Individualize clinical management to suit a patient's distinct characteristics and risk profile** SLE is a highly heterogeneous disease and patients vary widely with regard to disease course, disease manifestations, risk profile, priorities and preferences, tolerance to medications and response to specific treatments.

## Data Availability

No new data were generated or analysed in support of this research.
